# Validation of the OPTIMAL-Confidence Questionnaire in Patients with Chronic Low Back Pain

**DOI:** 10.3390/jcm14010221

**Published:** 2025-01-02

**Authors:** Sonia Nieto-Marcos, María José Álvarez-Álvarez, Iván Antonio Ramón-Insunza, Leonor García-Solís, María Mar Calvo-Arias, Arrate Pinto-Carral

**Affiliations:** 1 Complejo Asistencial Universitario de León, 24008 León, Spain; densnm00@estudiantes.unileon.es; 2 SALBIS Research Group, Department of Nursing and Physical Therapy, Faculty of Health Sciences, Campus de Ponferrada, Universidad de León, 24401 Ponferrada, Spain; apinc@unileon.es; 3 Primary Care Health Center of Ponferrada, Junta de Castilla y León Consejería de Sanidad, 47007 León, Spain; iaramon@saludcastillayleon.es (I.A.R.-I.); lgarciaso@saludcastillayleon.es (L.G.-S.); 4 Primary Care Health Center of Armunia, Gerencia de Atención Primaria de León, 24008 León, Spain; mcalvo@saludcastillayleon.es

**Keywords:** pain, chronic pain, evaluation, psychometrics, spine-low back, personalization

## Abstract

**Background/Objectives:** Low back pain is one of the most prevalent pathologies. Several studies relate its chronification to certain psycho-emotional characteristics, such as self-efficacy or the patient’s lack of confidence in the ability to move. Determine the reliability and validity of the OPTIMAL-confidence scale in people with chronic low back pain and describe the confidence in the movement capacity of this population. **Methods:** Design: A validation study was designed to evaluate the psychometric properties of the OPTIMAL-Confidence Questionnaire in a population with chronic low back pain. A descriptive observational study in a population with chronic low back pain and a healthy population was designed to describe the confidence in the movement capacity of the population with chronic low back pain. Settings: Health centers of primary care from a region of northwestern Spain. Participants: The final sample was 122 patients diagnosed with chronic low back pain. The sampling was completed with 30 additional healthy subjects. Instruments: OPTIMAL-confidence questionnaire, Numerical Pain Rating Scale, Chronic Pain Self-efficacy Scale, and ad hoc questionnaire to collect socio-demographic and clinical variables. **Results:** Cronbach’s alpha for the OPTIMAL-confidence questionnaire was 0.91. The association of OPTIMAL-confidence with the self-efficacy, pain intensity, and movement ability scales was moderate and significant (*p* < 0.001). Regarding the low back pain population, significant differences were observed in confidence levels according to age and the need for walking aids (*p* < 0.009). The OPTIMAL-confidence questionnaire also showed significant discrimination between the low back pain group and the no back pain group (*p* < 0.001). The confidence interval was 95%. **Conclusions:** The population with low back pain shows less confidence in their ability to perform movements, compared to the general population. OPTIMAL is an instrument that can discriminate between patients who present chronic low back pain and those who do not.

## 1. Introduction

Pain has been one of the most complicated challenges for healthcare professionals since ancient times. The word pain is used to refer to injuries or attacks to the body, although it can also be used to indicate experiences without external attribution, giving it an internal cause within the organism [1].

In 2020, the International Association for the Study of Pain (IASP) [2] proposed a new definition of pain as “an unpleasant sensory and emotional experience associated or similar to that associated with actual or potential tissue damage”.

Low back pain is one of the oldest and most prevalent musculoskeletal syndromes in living memory, affecting 80% of the population at some point in their lives. The disability associated with low back pain entails important health and labor costs, thus becoming a relevant socioeconomic problem [3,4]. In Spain, it is the cause of 11.4% of sick leave, representing a loss of 21.9 working days per worker per year and implying an expense to Social Security of some EUR 66 million per year [4].

We define low back pain as a musculoskeletal syndrome or set of symptoms characterized by pain, muscle tension, or stiffness focused on the final segment of the spine (lumbar area), in the area between the costal margins and the sacral region, and which can sometimes involve the gluteal region [3,5]. Depending on its duration, it is classified as acute low back pain (duration of less than three months) or chronic back pain (duration of more than three months and at least half of the days in the last six months) [6].

Most acute episodes resolve in less than two weeks, with a high percentage (30–60%) of relapses before the first year, and around one-third of these low back pain episodes become chronic [5,7].

Several studies [8,9] establish an association between chronic pain in general and low back pain in particular, with altered psycho-emotional states such as depression, stress, and anxiety, among others. The chronification of low back pain leads to a modification in the representation of pain at the cerebral level; thus, other areas, such as the cognitive and emotional areas, acquire greater prominence [10]. Frequently, these neuroplastic maladaptive conditions are influenced by dysfunctional beliefs, attitudes, and behaviors. Among the beliefs described as most important in chronic low back pain, self-efficacy [11] stands out [12].

The first references to the concept of self-efficacy were found in 1977, when it was introduced by Bandura [13], defining it as “people’s judgments about their abilities to reach certain levels of performance”. Several studies [14] have shown that self-efficacy is one of the most conclusive factors that influence the outcome of treatment in patients with chronic pain. This concept of self-efficacy [11] is strongly related to the construct of confidence, concluding that an outcome measure that could capture the impact of a person’s sense of confidence or control over the ability to perform activities would add an important dimension to the understanding of the problem.

Demonstrating that individuals with low back pain exhibit low self-efficacy is essential because self-efficacy plays a crucial role in pain perception and health-related behaviors. Self-efficacy reflects individuals’ confidence in their ability to handle challenging situations, such as pain [9]. In the context of low back pain, low self-efficacy can lead to increased movement avoidance, fear of pain, poor treatment adherence, and a vicious cycle of inactivity and disability.

The Outpatient Physical Therapy Improvement in Movement Assessment Log (OPTIMAL) questionnaire, developed by Guccione et al. [15] and validated in Spanish by Pinto Carral et al. [16], was established according to two scales: one that assesses the degree of difficulty associated with the limitations of the activity and another one that assesses the degree of confidence to perform the movements. The confidence scale is based on Bandura’s self-efficacy theory [11] on the role of the patient’s beliefs and sense of mastery in the execution of certain movements. Both scales have been validated using the PF10 scale of the SF36 [15,16] as a reference, although only the difficulty scale measures the physical construct of activity performance, whereas the confidence scale has not been correlated with an adequate construct. Therefore, it is essential to validate this confidence scale using another instrument that serves as a reference for measuring the same construct.

The OPTIMAL-confidence scale is used to assess patients’ perception of their ability to perform specific activities. Validating its use in individuals with chronic low back pain is essential to ensure its validity and reliability in this specific group. By validating this scale in this population, the goal is to provide clinicians and researchers with a reliable tool to evaluate and address key psycho-emotional aspects in the progression of patients diagnosed with chronic low back pain.

The lack of studies on this validation in the population with chronic low back pain, together with the importance of investigating all the factors that can act as catalysts of a state of chronification of the disease, generate the need to create instruments that allow assessing confidence in the movement of patients with chronic low back pain.

The study was designed with two distinct but complementary objectives, as follows:

- The first objective was to evaluate the reliability and validity of the confidence subscale of the OPTIMAL instrument in individuals with chronic low back pain. This involved a thorough psychometric analysis, including assessments of internal consistency and construct validity through comparisons with established measures;
- The second objective was to describe the levels of confidence in movement capacity within this population. This descriptive analysis aimed to provide a comprehensive characterization of the population’s confidence levels, enhancing the understanding of movement-related confidence in individuals with chronic low back pain.

By addressing these two goals, the study contributes both to the validation of the OPTIMAL instrument for clinical use and to a deeper insight into the movement confidence profile of this specific patient group.

## 2. Material and Methods

### 2.1. Design

A validation study was designed to evaluate the psychometric properties of the OPTIMAL-Confidence Questionnaire for a population of individuals with low back pain, previously translated and validated in Spanish. The study design was observational and cross-sectional, focusing on assessing the psychometric properties of the questionnaire. Reliability (internal consistency) and construct validity were evaluated through linear associations between the OPTIMAL-confidence scores and a validated self-efficacy questionnaire, pain intensity, and movement capacity.

A descriptive observational study in a population with chronic low back pain and a healthy population was designed to describe the confidence in the movement capacity of the population with chronic low back pain.

This study is registered on ClinicalTrials.gov (NCT05860426) to facilitate the potential implementation of future clinical trials addressing chronic low back pain in primary healthcare settings. By establishing this registry, we aim to contribute to the development of robust evidence-based interventions tailored to this prevalent condition, ensuring accessibility and practical applicability within primary care environments.

### 2.2. Study Setting and Sample

A sample was selected from patients assigned to primary care health centers from a region of León (Spain), who were diagnosed with chronic low back pain or chronic low back pain by their referring physician. It was completed with convenience sampling to select 30 subjects from the healthy population.

To calculate the optimal sample size, the criteria included in the COSMIN guide (consensus-based standards for the selection of health measurement instruments) [17] state that it should be equal to or greater than 100 subjects.

The scope of the study was five health centers belonging to the Regional Management of Primary Care of El Bierzo and León (Spain).

### 2.3. Inclusion and Exclusion Criteria

The study included adults (1) over 18 years of age, (2) in whom low back pain had been a problem for more than three months, (3) who had the ability to read and understand Spanish, and (4) who had signed the informed consent form [18].

The study excluded the candidates who refused to participate and/or presented any of the following pathologies: tumor and/or infection related to the back, cauda equina syndrome, vertebral compression fracture, abdominal aneurysm, central nervous system disorders, confirmed diagnosis of active neoplasia, pregnancy in the last year, and/or presence of cognitive deficits that prevented participation in the study [6,18].

### 2.4. Instruments

An ad hoc questionnaire was used to collect sociodemographic and health data (age, sex, country of birth, area of residence, marital status, educational level, employment status, weight, height, chronic diseases, treatments, weekly frequency of physical activity, assistance with ambulation, pain location, irradiation, numerical pain scale [19], and movement capacity scale [20]).

The numerical pain scale measured pain intensity using a scale from 0 (no pain) to 10 (maximum pain), where the subject responded to the following statement: “Indicate the number that best represents the intensity of your pain today” [19]

The quantification of movement capacity was carried out using a scale from 0 to 10, where 0 represents a “total lack of movement” and 10 represents a “world-class athlete” [16]. The subject responded to the following statement: “Indicate on this scale from 0 to 10 what you consider your movement capacity to be today”.

The OPTIMAL-confidence scale in Spanish consists of three subscales: upper limb (items 1 to 4, 6, 7, and 9), lower limb (items 5, 8, and 10 to 16), and trunk (items 17 to 22). The response options are scored from 1 (“I am totally confident in my ability”) to 5 (“I am not confident in my ability”). If any item is marked as “not applicable”, it does not score, thus the maximum score achievable is on the remaining number of items. With the sum of the total scores and using the formula indicated by the original authors of the instrument [15], we obtained, both for total score for the scale and for each of the subscales, a value from 0 to 100, with higher scores indicating lower confidence (greater disability).

The Chronic Pain Self-Efficacy Scale [21] was also used in its version validated in Spanish by Martín-Aragón et al. [22]; it has adequate internal consistency and test–retest reliability indices and was used as a comparison instrument to determine construct validity.

It consists of 19 items grouped into three categories: self-efficacy in symptom control, self-efficacy in physical functioning, and self-efficacy in pain management. The response options range from 0 (I believe myself to be totally incapable) to 10 (I believe myself to be totally capable), with the sum of the total scores on the 19 items giving a final value out of 100, where higher scores indicate greater self-efficacy.

### 2.5. Data Collection

Data collection was carried out by nurses and physiotherapists from the health centers belonging to the mentioned health areas, who were previously trained in meetings with the principal investigator.

Those who met the inclusion criteria were informed of the possibility of participating in the study freely and voluntarily, and those interested filled out the informed consent document.

### 2.6. Data Analysis

A descriptive analysis was performed for each of the sociodemographic and health variables collected in the questionnaire, using the observed frequencies with a 95% confidence interval [23]. Measures of central tendency were also calculated, such as mean and standard deviation for quantitative variables and median and interquartile range for quantitative variables whose distribution was significantly different from normality. Distribution and normality were determined by Kolmogorov–Smirnov tests. An initial comparison was made between the lumbar population group and the general population group using the Student’s *t*-test and chi-square tests.

The internal consistency of OPTIMAL, as well as that of each of its subscales, was determined using Cronbach’s alpha [24].

To determine the construct validity, the linear association between the results of OPTIMAL-confidence with those of the self-efficacy, pain intensity, and movement capacity questionnaires was analyzed using Spearman’s coefficient (rho), given the non-normal distribution of the main variables.

The validity of known groups was also analyzed by comparing the OPTIMAL-confidence results according to different pre-established groups (by age, walking aids, location of the pain, and presence or absence of low back pain). Given that the OPTIMAL results followed a non-normal distribution, for those cases in which two groups were compared, the Mann–Whitney U-test was used, and, for those cases in which three or more groups were compared, the Kruskal–Wallis H-test was used. In cases where there were significant differences using the latter test, subsequent pairwise comparisons were performed using the Mann–Whitney U-test [25].

All analyses were carried out with the IBM SPSS v26.0 statistical package. The significance level (*p*) was set at 0.05.

### 2.7. Ethical Considerations

Approval of the study was obtained through favorable reports from the Ethics Committee of the University of León of the University of León (ETICA-ULE-003-2021) and the Clinical Research Ethics Committee of the León Health Area (No. RI: 2131—30 May 2023). All participants signed an informed consent form, in accordance with the Declaration of Helsinki (Rev. 2013), and had the option to revoke their participation in the study at any time. The privacy rights of human subjects were observed. Ethical standards and the Data Protection Law (Organic Law 3/2018) and Law 14/2007 on Biomedical Research on Human Subjects were respected.

## 3. Results

### 3.1. Characteristics of the Sample

A total of 222 people with low back pain were invited to participate in the study, of whom 84 were excluded since they did not meet the established criteria, and another 16 candidates did not want to participate in the study. The remaining 122 agreed to participate and gave their written consent. Of the recruited patients, 84.4% belonged to the health area of El Bierzo, compared to 15.5% in the area of León.

The final sample consisted of 122 subjects with chronic low back pain and 30 subjects from the general population without low back pain. For eight individuals who explicitly refused to provide data related to their anthropometric characteristics (weight and height), these cases were considered as missing data. Where participants did not remember specific health-related information necessary to complete the questionnaire, their computerized medical records were consulted.

Of the total sample of 122 people with low back pain, 50 (41%) were men and 72 (59%) were women, with ages between 26 and 90 years (mean age ± SD = 58.61 ± 13.08).

Comorbidity was high (47% presented two or more chronic pathologies) and most of the participants (95.9%) were following some pharmacological treatment. A total of 61.5% reported having undergone physiotherapy treatment for their low back pain and 4.1% had received psychological support.

Specifically, the medications used in the treatment of low back pain were, in a percentage higher than 80%, analgesics and anti-inflammatory drugs. Corticoids were used less frequently (30.3% of the patients).

The subsample of the population without low back pain consisted of 30 individuals, 63.3% of whom were women and 36.7% men, with a mean age of 55.1 ± 13.06 years and a BMI of 25.68 ± 3.90. The percentage of individuals with chronic illnesses was 26.7%, and 90% of the sample was taking some form of medication for their low back pain, with analgesics being the most commonly used. Only 13.3% of the sample engaged in more than 10 h of physical activity per week. This population was similar to the low back pain population, with no significant differences for most of the socio-demographic variables (*p* < 0.05), except for the distribution by area of residence (*p* = 0.02), educational level (*p* = 0.04), body mass index (*p* = 0.04), and chronic diseases (*p* < 0.001).

Table 1 shows the main characteristics of the two samples studied, as well as the comparison between them.

Table 2 shows the results obtained from the OPTIMAL-confidence and self-efficacy scale scores, as well as their subscales, in the low back pain population.

**Figure 1:**
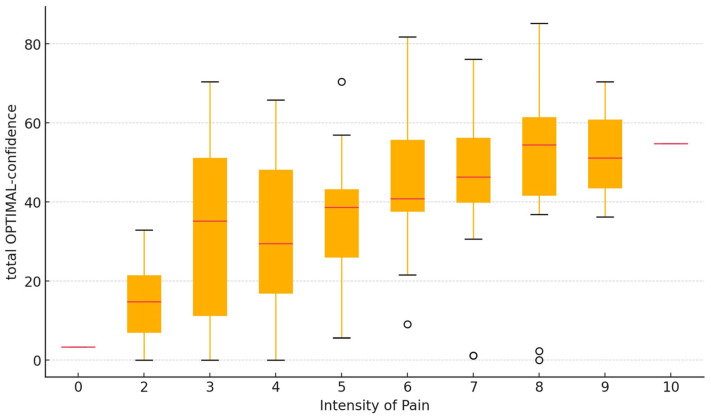
Association between total OPTIMAL-confidence and pain intensity. Small circles represent outliers.

**Figure 2:**
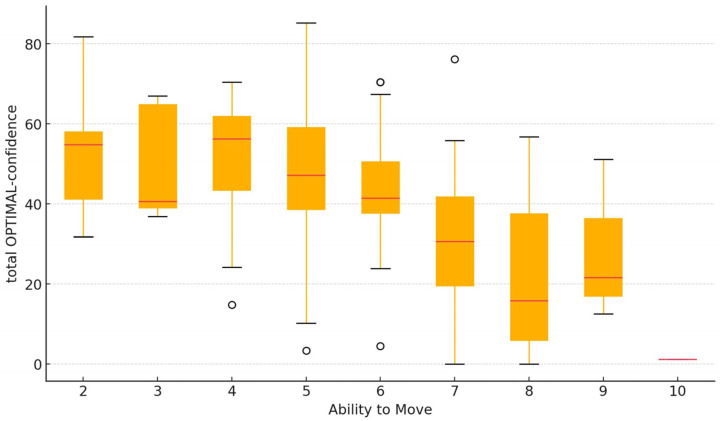
Association between total OPTIMAL-confidence and ability to move. Small circles represent outliers.

Figure 1 and Figure 2 illustrate the relationship between the total OPTIMAL-confidence score and two key clinical variables. Figure 1 depicts the association between total OPTIMAL confidence and pain intensity, highlighting variations in confidence across different levels of perceived pain. Figure 2 presents the relationship between total OPTIMAL confidence and the ability to move.

The OPTIMAL-total confidence scale obtained a 0% floor effect. The ceiling effect was 3.27%; thus, this percentage of patients indicated maximum confidence, i.e., minimum disability.

When completing the OPTIMAL-confidence questionnaire, patients indicated the “not applicable” option in a higher percentage for the items squatting (8.2%), kneeling (7.4%), and bending (3.3%).

Pain location was almost equally distributed among the three established regions (localized, radiating/referred, or generalized pain) (Table 3) with a median of 6 on both the pain intensity and movement ability scales (Table 2).

### 3.2. Psychometric Properties of the OPTIMAL-Confidence

The internal consistency through Cronbach’s alpha was high, being 0.91 for the total scale and higher than 0.8 in the subscales (trunk subscale: 0.86, lower extremity subscale: 0.81, and upper extremity subscale: 0.87) [26].

Regarding construct validity, the association between OPTIMAL confidence and self-efficacy was inverse (the higher the OPTIMAL score, the lower the self-efficacy score), moderate, and significant, with a rho value of −0.47 (*p* < 0.001).

The association between OPTIMAL confidence and pain intensity was direct (the higher the OPTIMAL score, the higher the pain intensity score), moderate, and significant, with a rho value of 0.44 (*p* < 0.001).

An association was found between OPTIMAL confidence and movement ability, which was inverse (the higher the OPTIMAL score, the lower the movement ability score), moderate, and significant, with a rho value of −0.52 (*p* < 0.001).

Table 3 shows the comparison of the OPTIMAL-confidence score as a function of different prespecified analysis groups.

Regarding the low back pain population, significant differences were observed (*p* = 0.009) in the OPTIMAL confidence results according to age, with less confidence being obtained in the older groups. In the post hoc analysis between groups, it was observed that the group of people under 50 years of age presented greater confidence with respect to the group of 62–67 years of age (*p* < 0.006) and with respect to those over 68 years of age (*p* < 0.003). Less confidence was also observed in patients requiring walking aids (*p* = 0.009). On the other hand, no significant differences were detected between groups according to the location of the pain (*p* = 0.147), although the group with generalized pain was the least confident, followed by the group with radiating/referred pain, as expected.

The OPTIMAL confidence questionnaire also showed the capacity to significantly discriminate between the low back pain group and the group without low back pain (*p* < 0.001).

## 4. Discussion

The main objective of this study was to determine the reliability and validity of the OPTIMAL confidence instrument validated in Spanish [16] in people with chronic low back pain.

The analyses of construct validity confirm the hypotheses proposed, with all associations being moderate and significant.

The OPTIMAL-confidence scale refers to the concept of confidence in the ability to perform movements, which is closely related to the construct of self-efficacy, understood as the beliefs and sense of mastery when moving [13]. Two previous studies relate high levels of self-efficacy to lower levels of disability caused by chronic low back pain [27,28]. Although both studies corroborate the mediation of self-efficacy together with fear of movement in relation to pain, self-efficacy proved to be the strongest mediator between pain intensity and disability in patients with chronic low back pain. Other authors [29,30] have shown that the use of techniques for the control of self-efficacy in pain management reduced the coefficient of the relationship between pain intensity and disability.

Along the same lines, previous studies [31] show that patients with chronic low back pain who were classified as having a low level of self-efficacy had shorter ranges of motion in the lumbar flexion gesture and lower postural stability. These results indicate that the level of self-efficacy not only affects the psychosocial sphere but can also interfere with physical performance and even result in functional limitation.

The intensity of the association between OPTIMAL confidence and pain intensity has also been found to be moderate and significant. Previous studies concur with these findings. Roy La Touche [31] shows that patients diagnosed with chronic low back pain who had a lower level of self-efficacy reported higher pain intensity during activity-related repetition processes. Martín-Aragón et al. [22] also corroborated this aspect by observing, in a pain management unit, that negative self-efficacy expectations and beliefs correlate with greater pain intensity in patients with benign chronic pain.

By analyzing the OPTIMAL confidence scale in more detail, we observed that the subscale that obtained the highest scores was the lower limb scale. This indicates a lower confidence of the subjects in their ability to execute lower limb movements. Kahraman [32] concluded that people who reported more severe low back pain had higher levels of lower limb disability. All these findings seem to indicate that this association is due to the fact that lower body movement exerts forces on the spine that can affect the lumbopelvic region. It has been shown that patients with low back pain often present pelvic misalignment, which is manifested by an asymmetrical posture of the lower part of the body, particularly unequal leg length and compensatory changes in the foot, that is, in the prone/supination direction [33]. Low back pain has also been related to muscle weakness in the buttocks, tightness in the hamstrings and iliacus, and tightness in the quadratus lumborum [34], as well as weaker lower abdominal muscles and limited range of motion in the hips [35,36]. These findings are compatible with the repercussions of the lower limbs on the statics and dynamics of the lumbar spine [37].

The validity of known groups allowed for determining the capacity of the OPTIMAL confidence questionnaire to differentiate between groups of people with different characteristics within the population with low back pain.

The analyses showed that, in the population with low back pain, the older groups had less confidence in their ability to move, which is in line with the results of a previous study that related low functional self-efficacy to the appearance of disabling musculoskeletal pain in patients over 70 years of age [38]. The same result has been obtained in the validity of groups established by the use of assistive devices for ambulation [39], where those who used some type of aid were less confident in their ability to move. Both results coincide with those obtained in 2019 in the Spanish validation study of the OPTIMAL questionnaire [16].

Patients with widespread pain had higher OPTIMAL-confidence scores than those with localized or radiating pain. The generalized and persistent nature of chronic pain forces patients to make continuous adjustments in order to learn to live with their disease, making self-efficacy a key factor in its management [40]. Along the same lines, other studies suggest that factors such as self-efficacy may be mediated by hypersensitivity induced by central sensitization [41].

Finally, the population with low back pain shows, as expected, less confidence in their ability to perform movements compared to the general population, and the differences are significant. Thus, OPTIMAL is an instrument that can discriminate between patients who present chronic low back pain and those who do not [42].

### 4.1. Limitations

The main limitation of this study is the large number of OPTIMAL-confidence items that participants marked as “not applicable”. This could be due to the fact that many people have personal beliefs or medical indications that they cannot perform a particular movement. Probably, the final score would have been higher if these patients had completed all the items.

### 4.2. Recommendations of Further Research

A fundamental aspect in the care of subjects with chronic low back pain will be the identification of cases with low levels of confidence since it is expected that this group of patients will be the greatest beneficiary of specific interventions. As future lines of research, clinical trials should be conducted to evaluate educational, cognitive, and behavioral therapies that demonstrate optimal results through good pain control, as well as improving patient confidence [29,30,43].

### 4.3. Implications for Policy and Practice

The results of this study provide information that can improve care for patients with chronic low back pain. Although fear of movement has received much attention in the literature related to low back pain [44], our study identifies confidence in the patient’s ability to move as an important key in the evolution of this pathology, even mediating its chronification. This suggests that it may be more effective to focus on therapies aimed at improving confidence, rather than only reducing pain.

These findings suggest several clinical implications that could enhance the management of chronic low back pain by addressing movement confidence. One potential approach is the promotion of progressive physical activity, designing exercise programs that gradually increase in load and complexity to help patients overcome fear of movement, while improving their confidence.

Another crucial intervention is therapeutic pain education, which helps patients reinterpret their symptoms as manageable and non-threatening rather than as signs of damage. Complementary strategies, such as movement desensitization using videos, demonstrations, or mental exercises, can also help patients build trust in their ability to move safely.

Finally, adopting a multidisciplinary approach, involving collaboration with psychologists to address limiting beliefs and occupational therapists to integrate movement strategies into daily tasks, provides a holistic framework. Together, these strategies highlight the importance of enhancing confidence as a cornerstone of effective chronic low back pain management.

Overall, the findings of this study highlight the validity of the OPTIMAL-confidence instrument for measuring confidence in the ability to move in patients with chronic low back pain.

## 5. Conclusions

The OPTIMAL-confidence instrument is presented as a reliable and valid self-reported instrument to measure the confidence that people with chronic low back pain have in their ability to move.

## Figures and Tables

**Table 1:** Descriptive data of the sample.

	Low Back Pain (N = 122) n (%)	CI ^§^ 95%	Healthy Population (N = 30) n (%)	CI ^§^ 95%	*p*-Value
Age †	58.61 ± 13.08		55.10 ± 13.06		0.19
Gender					0.67
Men	50(41)	(32.2–50.3)	11(36.7)	(19.9–56.1)	
Women	72(59)	(49.7–67.8)	19(63.3)	(43.9–80.1)	
BMI †	27.62 ± 4.96		25.68 ± 3.90		0.05
Area of residence					0.03
Rural	46(37.7)	(29.1–46.9)	5(16.7)	(5.6–34.7)	
Urban	76(62.3)	(53.1–70.9)	25(83.3)	(65.3–94.4)	
Marital status					0.37
Married	79(64.8)	(55.6–73.2)	18(60.0)	(40.6–77.3)	
Single	22(18)	(11.7–26)	9(30.0)	(14.7–49.4)	
Divorced	13(10.7)	(5.8–17.5)	1(3.3)	(0.1–17.2)	
Widowed	8(6.6)	(2.9–12.5)	2(6.7)	(0.8–22.1)	
Education					0.05
Primary	43(35.2)	(26.8–44.4)	3(10.0)	(2.1–26.5)	
High School/Vocational Training	58(47.2)	(38.4–56.8)	19(63.3)	(43.9–80.1)	
University	21(17.2)	(11–25.1)	8(26.7)	(13.3–45.9)	
Employment					0.50
Active	66(54.1)	(44.8–63.2)	16(53.3)	(34.3–71.7)	
Unemployed	8(6.6)	(2.9–12.5)	3(10.0)	(2.1–26.5)	
Retired	31(25.4)	(18–34.1)	10(33.3)	(17.3–52.8)	
Student	0(0)	(0–3)	0(0.0)	(0.0–11.6)	
Disabled	5(4.1)	(1.3–9.3)	0(0.0)	(0.0–11.6)	
Household chores	12(9.8)	(5.2–16.6)	1(3.3)	(0.1–17.2)	
Chronic diseases					<0.001
No disease	32(26.2)	(18.7–35.0)	22(73.3)	(54.1–87.7)	
One disease	32(26.2)	(18.7–35.0)	6(20.0)	(7.7–38.6)	
Two or more diseases	58(47.5)	(38.4–56.8)	2(6.7)	(0.8–22.1)	
Treatments performed for pain					0.10
No drug	5(4.1)	(1.3–9.3)	4(13.3)	(3.8–30.7)	
One drug	27(22.1)	(15.1–30.5)	4(13.3)	(3.8–30.7)	
Two or more drugs	90(73.8)	(65–81.3)	22(73.3)	(54.1–87.7)	
Frequency of physical activity					0.90
Less than 4 h/week	53(43.4)	(34.5–52.7)	13(43.3)	(25.5–62.6)	
Between 4 and 10 h/week	49(40.2)	(31.4–49.4)	13(43.3)	(25.5–62.6)	
More than 10 h/week	20(16.4)	(10.3–24.2)	4(13.3)	(3.8–30.7)	
Use of walking aid					0.15
Yes	15(12.3)	(7–19.5)	1(3.3)	(0.1–17.2)	
No	107(87.7)	(80.5–93)	29(96.7)	(82.8–99.9)	
Pain localisation					
Localised pain	44(36.1)	(27.6–45.3)			
Referred/irradiated pain	45(36.9)	(28.3–46.1)			
Generalised pain	33(27)	(19.4–35.8)			

^§^ CI: confidence interval. † Mean ± SD.

**Table 2:** Pain intensity, movement capacity, OPTIMAL-confidence, and self-efficacy instrument scores (N = 122).

Scales	Mean ± SD	Median (Q1–Q3)
Intensity of pain (0–10 scale)	5.61 ± 1.95	6 (4–7)
Ability to move (0–10 scale)	5.77 ± 1.78	6 (5–7)
Total OPTIMAL-confidence ^§^	39.56 ± 20.70	40.90 (24.09–55.04)
Trunk subscale	32.61 ± 23.11	32.14 (13.39–50)
Lower extremity subscale	44.00 ± 23.51	46.68 (30.23–63.88)
Upper extremity subscale	41.15 ± 24.10	41.66 (25–61.53)
Total Self-efficacy †	65.70 ± 17.38	68.42 (54.47–79.60)
Symptom Management	67.61 ± 16.86	68.75 (58.75–81.25)
Physical Functioning	74.78 ± 21.52	79.16 (63.33–91.66)
Pain Management	51.77 ± 21.99	56 (35.50–68)

^§^ Score out of 100, with a higher percentage corresponding to lower confidence. † Score out of 100, with a higher percentage corresponding to higher self-efficacy.

**Table 3:** Comparison of the results of OPTIMAL-confidence as a function of the sociodemographic characteristics and the analysis groups.

Analysis Group	OPTIMAL-Confidence Median (Q1–Q3)	*p*-Value
Age (N = 122)		
≤50 years (n = 37)	26.14(3.98–48.29)	0.009 **
51–61 years (n = 24)	40.91 (23.86–55.64)	
62–67 years (n = 31)	42.05(31.82–56.82)	
≥68 years (n = 30)	44.70(38.35–55.96)	
Use of walking aid (N = 122)		
Yes (n = 15)	55.68(39.77–64.77)	0.009 *
No (n = 107)	39.77(22.73–53.41)	
Pain localisation (N = 122)		
Localised pain (n = 44)	36.60(19.88–51.41)	0.147 **
Referred/irradiated pain (n = 45)	42.05(30.68–58.52)	
Generalised pain (n = 33)	43.95(27.84–56.90)	
Low back pain		
Yes (n = 122)	40.91(24.09–55.01)	0.001 *
No (n = 30)	10.23(0.85–23.01)	

* Significance test using the Mann–Whitney U-test. ** Significance test using the Kruskal–Wallis H-test.

## Data Availability

The data supporting the reported results are not publicly available due to privacy restrictions.
